# Bayesian Space–Time Analysis of Brain Cancer Incidence in Southern Ontario, Canada: 2010–2013

**DOI:** 10.3390/medsci7120110

**Published:** 2019-12-14

**Authors:** Ravi Ancil Persad

**Affiliations:** Independent Researcher, Toronto, ON M2J 4E2, Canada; persad_ra@outlook.com

**Keywords:** Bayesian, space–time, spatio-temporal, risk, cancer, obesity, head injury, Canada

## Abstract

Canada has one of the highest incidence rates of brain cancer in the world. This study investigates the space–time variation of brain cancer risk across Southern Ontario, Canada. A Bayesian spatio-temporal regression model is used to estimate the relative risk of brain cancer in the 12 spatial health units of Southern Ontario over a four-year period (2010–2013). This work also explores the association between brain cancer and two potential risk factors: traumatic head injury (THI) and excess body fat (EBF). Across all areal units from 2010–2013, results show that the relative risk of brain cancer ranged from 0.83 (95% credible interval (CI) 0.74–0.91) to 1.26 (95% CI 1.13–1.41). Over the years, the eastern and western health units had persistently higher risk levels compared to those in the central areas. Results suggest that areas with elevated THI rates and EBF levels were also potentially associated with higher brain cancer relative risk. Findings revealed that the mean temporal trend for cancer risk progression in the region smoothly decreased over time. Overall, 50% of the health units displayed area-specific trends which were higher than the region’s average, thus indicating a slower decrease in cancer rates for these areas in comparison to the mean trend.

## 1. Introduction

Brain cancer is classified as one of the most lethal and debilitating forms of cancer [1]. It is of significant concern in Canada owing to high mortality rates associated with the disease and low life expectancies upon diagnosis [2]. From a global perspective, Canada has one of the highest brain cancer incidence rates in the world [3]. An improved understanding of brain cancer incidence and its risk from both a geographical and temporal perspective is critical for disease monitoring and planning of public health programs, particularly for large populations in developed countries such as Canada.

The province of Ontario comprises of two main regions, Northern and Southern Ontario. Our study focuses on Southern Ontario, which is the more densely populated and urbanized region of the province [4]. Southern Ontario has a high prevalence of traumatic head injury (THI) and overweightness/obesity rates in many of its cities [5,6]. Our proposed population-based study presents an investigation on the ecological relation between THI and excess body fat (EBF) with brain cancer, hence we highlight some critical aspects of prior related epidemiological studies and their findings.

THI is commonly caused from accidental events such as falls and vehicular collisions [5]. The causative role of previous cranial trauma in relation to the occurrence of cancerous/malignant brain tumors has been a contentious matter [7]. For instance, population studies by Zampieri et al. [8] and Nygren et al. [9] found that head injuries were not positively correlated with cancerous brain tumors. The study by Zampieri et al. [8] focused on a minimal population sample size of 195 individuals with cerebral tumors within a small region of Italy. In addition to head trauma, their research explored the association between the cancerous tumors and selected risk factors including blood type, exposure to radiation, smoking, and alcohol habits. Notably, the study did not yield any relevant association between brain cancer and any of the evaluated risk factors. Nygren et al. [9] analyzed a Swedish cohort of 311,006 patients amongst which 281 persons were diagnosed with brain tumors. Based on an age, gender, and injury severity stratified Poisson regression analysis, they reported a null association between brain injury and tumor formation. Contrarily, other epidemiological studies suggest that patients who suffered from THI were at an increased risk of developing brain cancer compared to persons without any such prior conditions [10,11]. Using a stratified longitudinal regression analysis based on factors such as age, gender, and geographic region, Chen et al. [10] investigated the relation between THI and the onset of brain cancer amongst 5007 patients diagnosed with THI over a three-year period in Taiwan. A non-THI cohort comprising approximately 25,000 persons were also monitored for comparison. Their findings showed that those with THI were at a risk of forming malignant brain tumors approximately 4.67 times higher compared to non-THI persons. However, due to known epidemiological fallacies such as the possibility of different results owing to variation in geographic and demographic characteristics, Chen et al. [10] acknowledged that it is quite probable that their results were not generalizable and only applicable to the Taiwanese population.

Similar to THI, past ecological research has shown varied association between EBF and brain cancer. Gliomas are the most common form of malignant brain tumors [12,13]. Amongst an adult cohort from the United States comprising 51,529 males and 121,701 females, Cote et al. [14] utilized a Cox proportional-hazards model to investigate the association between glioma risk and several body characteristic predictors including height, waist circumference, body mass index (BMI), and body somatotype. Overall, their results indicated that BMI and waist circumference did not influence the formation of gliomas. However, persons with greater height levels were linked to increased glioma risk. In an epidemiological study amongst 12,488 glioma patients of European descent, Disney-Hogg et al. [12] analyzed the potential risk of several genetically-based obesity-related factors and glioma formation. These included BMI, type-2 diabetes, insulin resistance, and cholesterol levels. They did not find any positive association between glioma development and obesity-related factors. However, Møller et al. [15] and Wolk et al. [16] reported otherwise, both linking higher BMI to elevated brain cancer risk. These studies were based on population cohorts from Denmark (43,965 patients) and Sweden (28,129 patients), respectively. Both of these studies assessed obesity and its role in the risk for brain cancer, as well as other forms of cancers, including those of the liver, colon, and pancreas. Cancer risk was estimated using a standardized incidence ratio statistical analysis in both studies. Notably, Møller et al. [15] presented results which showed a four-fold increase in brain cancer risk among patients diagnosed with obesity. However, even through the use of longitudinal monitoring of patients, they were unable to identify and speculate on the potential mechanism(s) which provide an explanation of the relation between obesity and increased brain cancer risk.

In the recent past, research in the geographical sciences, specifically in the fields of spatial and spatio-temporal epidemiology, have provided important insights for the improved understanding of brain cancer [17,18,19]. López-Abente et al. [17] used a Bayesian-based Poisson spatial model for investigating the geographical distribution of brain cancer incidence in the municipalities of two Spanish regions, Navarre and the Basque country. A primary objective of their study focused on environmental risk factors and in particular, the influence of explanatory land use variables on the disease. Socio-economic covariates such as unemployment and illiteracy were also analyzed but showed no association with brain cancer variation in the region. However, their findings showed that areas with a high percentage of non-irrigated arable land were at an increased risk of brain cancer. Lawson and Rotejanaprasert [18] used a Bayesian spatial modeling approach for the analysis of childhood brain cancer in Florida from 2000 to 2010. Specifically, a zero-inflated Poisson model was used owing to the rarity of childhood brain cancer and the capability of the zero-inflated distribution to handle the modeling of areal units with null cases. Their study did not have any covariates in the modeling process as case counts were accumulated for the 10-year period and used for hot-spot clustering risk analysis. Ugarte et al. [19] conducted a space–time analysis of brain cancer in Spain from 1986 to 2008 using a non-parametric Bayesian model. Explanatory covariates were not included in their modeling process as their primary objective concentrated on disease surveillance (i.e., identifying the spatio-temporal pattern of brain cancer incidence and the high/low risk areas within the study region). In a related study, Etxeberria et al. [20] jointly investigated both brain cancer incidence and brain cancer mortality using a Bayesian-based age–gender shared component model for regions in Spain. A shared component approach was used since it can handle the simultaneous modeling of both incidence and mortality data from the disease. Their methodology focused on analyzing the spatial patterns of the disease from 1990–2008. A common theme in these studies was the utilization of Bayesian hierarchical modeling (BHM). This statistical modeling approach has been widely applied for the analysis of both infectious and non-infectious diseases [21]. BHM is well suited for analyzing geo-referenced disease data since it enables: (i) parameter modeling and estimation through regression analysis for both fixed effects (e.g., covariates such as disease risk factors) and random effects (e.g., spatial clustering and space–time interaction effects); (ii) probabilistic inference (i.e., the use of probability distributions to compute parameters and their uncertainty); and (iii) the inclusion of prior knowledge for parameter estimation [22].

In our proposed study, a spatio-temporal regression analysis based on BHM is used to understand the underlying space–time distribution of brain cancer risk across Southern Ontario, Canada during a four-year span from 2010 to 2013. Moreover, during this period, we also aim to explore and determine the spatio-temporal association between brain cancer and two potential risk factors—THI and EBF—for the study region. Overall, the association between EBF and THI with brain cancer remains obscure and inconsistent amongst existing studies. From an epidemiological standpoint, this can be attributed to the generalization difficulties faced by observational population studies where the exposure to causative disease factors differ from one geographical location to the next, as well as temporally, from one period to the next [23]. Despite these practical limitations, localized epidemiologic research can elucidate important insights for regional monitoring of the disease. To the best of our knowledge, our presented work is the first to investigate the impact of both EBF and THI on brain cancer for the local region of Southern Ontario, Canada from a Bayesian spatio-temporal perspective. Thus, we view this study as an important evaluation on whether both can potentially be considered as brain cancer risk factors for the region during the assessed four-year time frame.

## 2. Materials and Methods

### 2.1. Datasets

**Figure 1:**
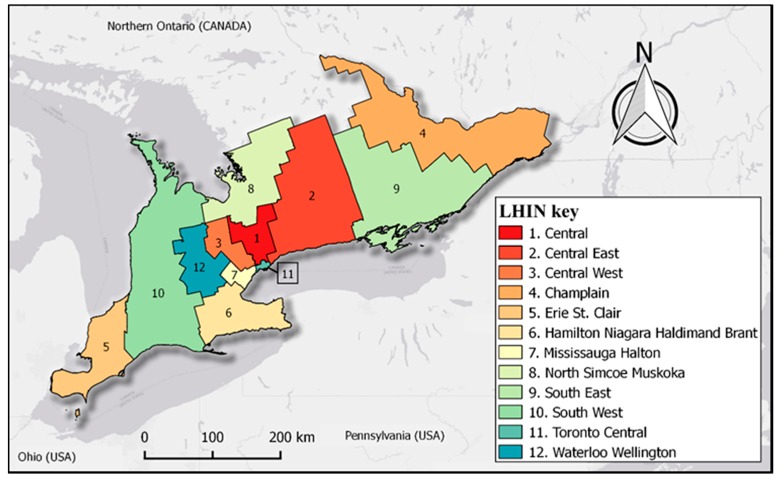
Map of study area showing the locations of the Local Health Integration Networks (LHINs) across Southern Ontario, Canada.

This study is based on the spatial (geographic/areal) units of 12 Local Health Integration Networks (LHINs) in Southern Ontario [24]. The LHINs are regional administrative bodies responsible for managing healthcare in the Canadian province. LHINs are illustrated in Figure 1.

We use count data for individuals diagnosed with brain cancer in each of the following calendar years: 2010, 2011, 2012, and 2013. This dataset was obtained from Cancer Care Ontario and based on diagnosed person counts from each of the 12 LHINs who were aged 15 years and older [25]. The count data is the response/dependent variable in our regression model. In addition, for each LHIN, two explanatory variables (i.e., covariates) were used in the model: (i) percentage of the population who had excess body fat levels (i.e., persons with BMI ≥25 kg/m^2^) and (ii) crude rate of persons (i.e., number of persons per 100,000) who were hospitalized for traumatic head injuries. Table 1 provides summary statistics of the population, as well as those of the dependent and independent variables across all LHINs from 2010–2013.

The EBF data was sourced from the Canadian Community Health Survey (CCHS) of 2013 and obtained from Cancer Care Ontario [25]. The CCHS is based on health status data collected from persons residing in the various health regions within the provinces of Canada and covers approximately 98% of the Canadian population excluding individuals such as those who are full-time members of the Canadian Forces and persons on Indian Reserves and on Crown lands [26]. The CCHS was a multi-stage sample survey and had an average response rate of 62.2% across LHINs in Southern Ontario [26,27].

For spatio-temporal regression models, covariates can be modeled as globally constant (does not vary over space or time), spatially constant (i.e., independent variable changes over each time point only), temporally constant (i.e., independent variable changes over each areal unit only) or varying over both space and time [28]. Owing to data availability for only one calendar year, the EBF covariate is treated as a temporally-constant variable in our spatio-temporal regression model. Regarding THI, we used data from Public Health Ontario [29]. The THI covariate is treated as a space–time variable since annual datasets were available for each space–time combination.

Previous research has shown that time is an important factor in explaining the causal relation between THI and brain cancer. Tyagi et al. [30] elucidated that there is a general period of one year between experiencing a severe brain injury and the appearance of a brain tumor. Hence, in this study we lagged the THI by one year, for example, brain cancer incidence data in 2010 corresponds to THI rates from 2009 in the regression model.

To provide an indication on the level of heterogeneity across all 12 LHIN regions, we refer the reader to the (supplementary material Figure S1) showing plots with the variation of brain cancer counts, THI rates, and EBF percentages.

### 2.2. Spatio-Temporal Bayesian Modeling

Brain cancer is a relatively uncommon disease [20]. A Poisson distribution assumption is well suited for the statistical modeling of rare diseases with low counts amongst large populations [21]. In this work, we adopt the spatio-temporal Poisson regression model of Bernardinelli et al. [31]. Bernardinelli’s model was used since it is able to provide various insights on disease risk, including the mean time trend of the study area and area-specific time trends [32]. In addition, given that our study period only spans across four years, we use this parametric, Bayesian hierarchical model since it has been previously shown to be appropriate for the analysis of spatio-temporal datasets with relatively short time periods of less than five years [32,33].

Given the study region comprising N LHIN areal units, we have the observed counts of diagnosed brain cancer cases $O_{it}$ for a period of T years (where i= 1, 2, … N and t = 1, 2, … T). The Bayesian hierarchical model has three main levels. In the first level, the Poisson data likelihood is defined (Equation (1)). The term $E_{it}RR_{it}$ in Equation (1) is the computed brain cancer incident count after the model has been fit where, $E_{it}$ is the expected number of cancer cases for each LHIN i at each time period t and $RR_{it}$ is the relative risk of brain cancer incidence for each LHIN i at each time period t. $E_{it}$ is computed using Equation (2) where $\bar{R}$ is the overall brain cancer incidence rate for the entire region and $\rho_{it}$ is the population of each LHIN i at time t [21].

(1) $$O_{it}\;\sim \;Poisson\left(E_{it}RR_{it}\right)$$

(2) $$E_{it}=\rho_{it}.\bar{R}=\rho_{it}.\;\left(\frac{\sum_{i=1}^{N}\sum_{t=1}^{T}O_{it}}{\sum_{i=1}^{N}\sum_{t=1}^{T}\rho_{it}}\right)$$

$RR_{it}$ is expressed as a function of the EBF and THI covariates, as well as spatial and temporal components (Equation (3)). Temporal and spatial (structured and unstructured) random effects were included in the model as proxy variables to any unobserved factors which influence the risk estimation of brain cancer [34].

(3) $$RR_{it}=exp\left(\alpha +\;U_{i}+V_{i}+\beta_{THI}X_{it\left(THI\right)}+\beta_{EBF}X_{i\left(EBF\right)}+\gamma time_{t}+\delta_{i}time_{t}\right)$$

where, α is the model intercept, $U_{i}$ is a spatially structured (or spatial clustering) random effect term, $V_{i}$ is a spatially unstructured (or uncorrelated heterogeneity) random effect term, β terms are the regression coefficients for the THI and EBF covariates, X are the values of the two respective covariates, γ is the mean linear time trend at $time_{t}$ and $\delta_{i}$ is a random effect term for space–time interaction (i.e., temporal trend effects for LHIN areal units).

In the second level of the Bayesian hierarchical model, the stochastic $RR_{it}$ parameters are assigned prior distributions. Following Lawson et al. [22] and Law and Haining [35], essentially non-informative Gaussian prior distributions were specified for the γ and β parameters: $\gamma ,\;\beta_{THI},\;\beta_{EBF}\;\sim \;Norm\left(0,\;0.0001\right)$, whilst an improper uniform prior distribution was used for the intercept: α ~ dflat(). For both $U_{i}$ and $\delta_{i}$, the conditional spatial autoregressive (CAR) prior distribution was used since it ‘borrows strength’ from adjacent areal units for smoothing the risk estimation [36]. We refer to the work of Besag et al. [37] for further details on the CAR prior, a spatial distribution model that has been extensively applied in spatial analysis research. $V_{i}$ was set to a zero mean Gaussian distribution: $V_{i}\;\sim \;Norm\left(0,\sigma_{V}^{2}\right)$ and in the third and final level of the hierarchical model, gamma prior distributions were defined for the variances of the random effects (i.e., the hyperparameters of $U_{i}$, $V_{i},$ and $\delta_{i}$): $1/\sigma_{U}^{2},\;1/\sigma_{V}^{2},1/\sigma_{\delta }^{2}\sim Gamma\left(0.5,\;0.0005\right)$ [22,38,39]. For Bayesian modeling and analysis, we used the R programming language (version 3.4.2), the WinBUGS software (version 1.4) [40] and the R2WinBUGS package (version 2.1-21) [41] (note: all distributions displayed are represented in the WinBUGS programming syntax [42]).

Markov chain Monte Carlo (MCMC) simulation was used for model fitting [43]. MCMC simulation estimates the posterior distribution of the various, unknown relative risk parameters. To fit the model and to make subsequent inferential analysis, we generated three Markov chains at 500,000 iterations each, discarding the first 50,000 as the non-stationary, “burn-in” period. To assess model convergence, we visually inspected parameter trace plots which showed the samples of each chain [21]. Additionally, we checked the Brooks–Gelman–Rubin diagnostic, which was close to 1.0 for all parameters, thus indicating convergence of the multiple Markov chains [44].

The quality of regression model fitting to the observed data was assessed using the deviance information criterion (DIC). DIC is a Bayesian measure of evaluating the model’s quality, where lower values of DIC indicate a closer model fit to the data [45,46]. Moran’s space–time index ($Moran_{STI}$) was used to quantify spatio-temporal residual autocorrelation in the model residuals (Equation (4)). The model residuals ($y_{it}$) are the differences between the observed ($O_{it}$) and fitted ($E_{it}RR_{it}$) values of the brain cancer incidence count response variable. We used Griffith’s formulation of $Moran_{STI}$ [47], which is a combination of the Durbin–Watson statistic (commonly used for quantifying temporal autocorrelation) with the well-known Moran’s I coefficient (commonly used for computing spatial autocorrelation). Similar to the spatial Moran’s I, $Moran_{STI}$ also ranges between −1 and +1, where 0 indicates spatial randomness.

(4) $$Moran_{STI}=\frac{\left(NT-N\right)\cdot \;\sum_{t=2}^{t=T}\sum_{i=1}^{i=N}\sum_{j=1}^{j=N}w_{ijt-1}\cdot \;\left(y_{it}-\bar{y}\right)\left(y_{jt-1}-\bar{y}\right)\;}{\sum_{t=2}^{t=T}\sum_{i=1}^{i=N}\sum_{j=1}^{j=N}w_{ijt-1}\cdot \;\sum_{t=1}^{t=T}\sum_{i=1}^{i=N}{\left(y_{it}-\bar{y}\right)}^{2}\;}.$$

where, $w_{ijt-1}$ is a spatio-temporal adjacency weight matrix representing the relation between neighboring LHIN areal units and $\bar{y}=\sum_{t=1}^{t=T}\sum_{i=1}^{i=N}\frac{y_{it}}{NT}$. We note that $w_{ijt-1}=w_{ij}$ due to the temporal invariance amongst adjacent regions of the LHIN areal units [48].

## 3. Results

### 3.1. Model Fitting and Prior Sensitivity Analysis

In this study, two models were fit: Model 1, which is the spatio-temporal model without any covariate fixed effects and Model 2, which is the ‘full’ model including both fixed and random effects. We obtained a DIC value of 356.9 for Model 1 whilst Model 2 produced a marginally lower value of 353.2. Overall, the quality of fitting amongst the tested models were very similar in terms of DIC units, however, Model 2 was selected as our final model since it provides insights into spatially unstructured and structured random effects, fixed effects, as well as temporal trends and space–time random effects. (Note: We refer the reader to Figures S2–S4 in the supplementary material where we also provide a brief note on the spatial patterns of relative risk explained by the fixed and random effects).

To further investigate the quality of Model 2, we examined the spatio-temporal autocorrelation in the model residuals. A spatio-temporal model with close fit to the data should have low spatio-temporal autocorrelation in its residuals, which is indicative of a good model [49]. For Model 2, we obtained a $Moran_{STI}$ value of 0.009, which implies that there is minimal space–time autocorrelation in the residuals.

For Bayesian inference, the setting of prior parameters is an important aspect. We conducted a sensitivity analysis with respect to the prior distributions of the regression coefficients β and γ, as well as the priors of the hyperparameters σ for the random effects U, V, and δ. With the lack of any actual prior expectations pertaining to the magnitude and direction of the regression coefficients, we follow Law and Haining [35], who recommend a normal distribution with zero mean and a large variance. Similar to Law and Haining [35], we tested the sensitivity of the regression coefficients when fitted with a noninformative prior: Norm(0, 0.0001), as well as with a more informative prior: Norm(0, 0.1). Following Jang et al. [39], we also assessed the sensitivity of two commonly-used priors for random effects: Gamma(0.5, 0.0005) and Gamma(0.001, 0.001). The former was recommended by Kelsall and Wakefield [50], whilst the latter is often applied in the WinBUGS software [51]. In addition, we also evaluated a uniform distribution prior for the standard deviations of the random effects: $\sigma_{U},\;\sigma_{V},\;\sigma_{\delta }^{\;}\sim Unif\left(0,\;1000\right)$ [21]. Following sensitivity testing, we found that the different prior selections for both the regression coefficients and hyperparameters of the random effects produced very similar posterior distributions of the parameters. We selected the prior Norm(0, 0.0001) for the regression coefficients and the prior Gamma(0.5, 0.0005) for the hyperparameters of the random effects owing to lower model fitting DIC values compared with their respective alternatively tested priors. However, we note that the differences in DIC values were not very significant (i.e., there was a maximum difference of 2.3 DIC units between the selected priors and all other evaluated priors). In addition, there was very minimal change in the relative risk of the individual LHIN areal units when using different priors (see plots, Figures S5–S8). Overall, sensitivity analysis showed that the parameter results were robust to the choice of priors which were assessed.

### 3.2. Spatio-Temporal Analysis

The results of the spatio-temporal Bayesian regression are presented in Table 2. From the posterior distribution of each estimated parameter, we obtained several statistics, including the parameter’s mean, standard deviation (SD), 95% credible interval, and the Monte Carlo (MC) error. We used the posterior mean as a point estimate for each parameter and the posterior standard deviation as a measure of parameter uncertainty. The 95% credible interval gives an indication of the range in which the parameter lies at a probability of 95%. MC error provides a measure of parameter accuracy and as a rule of thumb should be less than 5% of the parameter’s standard deviation [52]. The MC errors of all estimated parameters for this study were below 5% of their corresponding standard deviations.

To consider the relevance of both risk factor covariates on brain cancer incidence, the posterior probability of the β coefficients being different from zero was estimated (i.e., the probability of the coefficients being positive or negative). The posterior probability is considered to be a Bayesian counterpart of the *p*-value which is commonly used for evaluating the statistical significance of parameters in frequentist models [32,53]. To indicate parameter significance in this study, we followed previous research which have utilized the posterior probability of the parameter being different from zero (i.e., the posterior probability of the parameter being either positive or negative is greater than 90%) [54,55]. We found that the probability of being positive for EBF and THI was both higher than 90% and thus relevant to the response variable, specifically, $p\left(\beta_{EBF}>0|O_{it}\right)=$ 91.6% and $p\left(\beta_{THI}>0|O_{it}\right)=$ 99.7%.

The signs of regression coefficients for explanatory variables describe their effect on the response variable. From Table 2, both $\beta_{EBF}$ and $\beta_{THI}$ show a positive association with brain cancer. Hence, LHINs with higher levels of obesity/overweightness and greater rates of hospitalization due to traumatic head injuries tend to have higher associated relative risk of brain cancer in Southern Ontario. The relative influence of EBF and THI on brain cancer risk can be expressed as exp(β. ΔX). For instance, a 5% rise in EBF levels of a LHIN increases the relative risk by 21% and similarly, a 3 unit rise in the THI rate of a LHIN increases its relative risk by 28%. Table 2 also shows the results for the variances of the random effects, $\sigma_{U}$, $\sigma_{V}$, and $\sigma_{\delta }.$ The magnitude of these variances provide a general indication of risk structure [37]. Hence, amongst all three random effects, spatial structure U (i.e., effects due to spatial clustering) had the highest influence on the disease risk.

**Figure 2:**
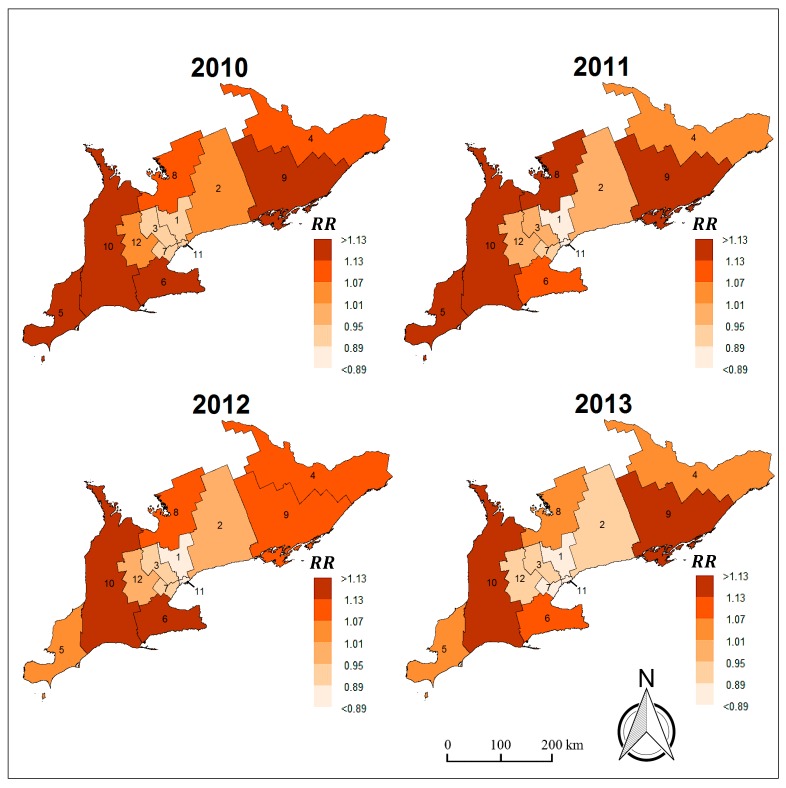
Relative risk maps of brain cancer for Southern Ontario, Canada (2010–2013).

Figure 2 illustrates the spatio-temporal relative risk of brain cancer incidence in Southern Ontario LHINs from 2010 to 2013, where brighter regions represent lower associated risk and darker areas indicate higher risk. If the relative risk of a LHIN is greater than 1.0, this implies that the population in the LHIN is at an increased risk of developing brain cancer due to an incidence frequency which is higher than the study area’s average. Similarly, LHINs with a relative risk below 1.0 are associated with lower brain cancer risk due to incidence frequencies which are lower than the study area’s average [56]. For instance, it is noticeable that the South West LHIN (ID. 10 in Figure 2) has relative risks which are greater than 1.0 across multiple years, specifically, with a minimum relative risk of 1.14 in 2013 and a maximum relative risk of 1.20 in 2010. Therefore, it can be inferred that the population in the South West LHIN had a risk of developing brain cancer which was 20% higher compared to the entire region in 2010 and 14% higher compared to the region in 2013.

The South East LHIN (ID. 9) had the highest cancer risk for a single year across the four-year period, with a relative risk of 1.25 in 2010. The Central LHIN (ID. 1) had the lowest annual cancer risk of 0.82 in 2013 (i.e., the population in the Central LHIN was 18% less likely to develop brain cancer compared to all other LHINs in 2013). Generally, the areal units in the eastern and western ends of the region had consistently higher risk levels compared to the centrally located LHINs over the years.

**Figure 3:**
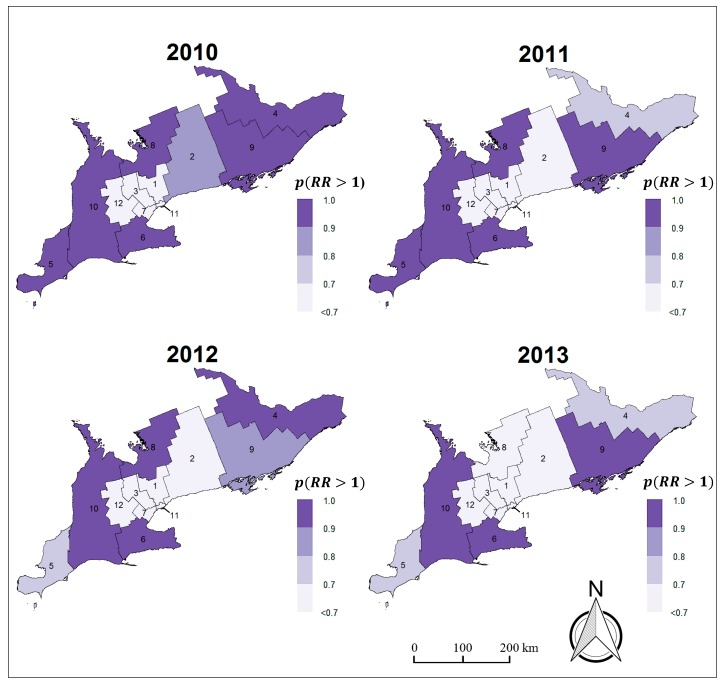
Exceedance probability maps of brain cancer for Southern Ontario, Canada (2010–2013).

Overall, the risk of developing brain cancer is smoothly decreasing over time in the Southern Ontario LHINs. This is visually evident from the maps in Figure 2 where the shades of several LHINs become increasingly brighter from one year to the next at various instances during the four-year period. This decrease in risk over space and time is also reflected by the negative coefficient of the mean linear time trend parameter γ (Table 2), where the posterior probability of γ being negative was greater than 90% and thus considered relevant to the outcome variable: $p\left(\gamma \langle 0|O_{it}\right)=$ 92.7%. Figure 3 illustrates the exceedance probabilities for LHINs (i.e., areal units where the posterior probability of brain cancer risk is greater than 1: $p(RR_{it}>1|O_{it}))$.

**Figure 4:**
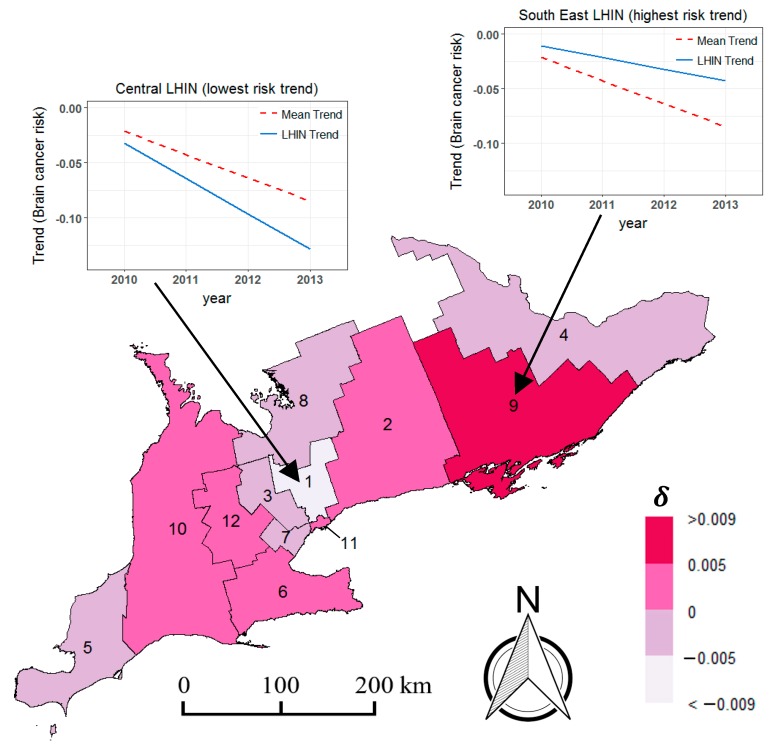
Differential trends of brain cancer risk for Southern Ontario LHINs, 2010–2013.

The space–time random effect δ is equivalent to the difference between the temporal trend of an individual LHIN and the mean trend γ of the entire region [31]. Figure 4 illustrates the distribution of these differential trends ($\delta^{\prime }s$) for all LHINs. When δ>0, this is indicative of a LHIN unit with a temporal trend that is higher than the corresponding mean trend for the entire study area. Likewise, when δ<0, this suggests that the temporal trend of the LHIN is lower than the overall mean trend. As shown by the trend plots in Figure 4, the Central LHIN had the lowest temporal trend whereas, the South East LHIN had the highest temporal trend. The temporal trend provides insight on the relative speed of cancer risk progression in the various areal units [57]. Since the South East LHIN had the highest trend, this implies that the risk for this area progressed the fastest in comparison to both the overall mean trend, as well as the local trends of all other LHINs. In contrast, given that the Central LHIN had the lowest trend, it can be inferred that disease risk for this health unit progressed the slowest over the period of study. As illustrated in Figure 4, six of the twelve LHINs (i.e., IDs 2, 6, 9, 10, 11, 12) had local trends which were greater than γ.

## 4. Discussion

Our results have shown that in Southern Ontario between 2010–2013, there was a positive association between brain cancer risk and two health-related factors, namely traumatic injuries to the head and high body fat levels. Regarding THI, our findings were in accordance with previous studies from the medical sciences where severe head injuries were found to be a potential predisposing factor for the onset of brain cancer [58,59,60]. From a neurological perspective, Spallone et al. [7] explained that cancerous brain tumors are commonly formed in the anatomical vicinity of sites where previous head injuries have occurred. Salvati et al. [61] expanded on this further by explaining that the presence of intracranial trauma causes a carcinogenic effect, thus promoting the rate of cancer formation in the brain.

The results of our study are also consistent with preceding epidemiological research. Both Chen et al. [10] and Hu et al. [11] conducted population-based studies and suggested a possible etiological relationship between THI and brain cancer. Further support was also presented in other work by Wee et al. [62] who applied a regression analysis on a cohort of 34,556 patients with THI and 69,112 patients without THI over a period of 15 years in Taiwan. Their results showed that brain cancer incidence was higher in patients with THI (4.38%) in comparison to those without THI (3.88%). THI is categorized as an unintentional injury caused by a forceful direct blow to the head, neck or facial regions of the body and as explained by Rao et al. [63], unintentional injuries are the fifth leading cause of deaths in Canada. Therefore, it is very possible that the precise estimation of brain cancer risk amongst provincial Canadian populations is distorted due to associated high mortality rates affecting persons with THI (i.e., death occurs before potential brain cancer formation). In terms of geographical similarity, a dated epidemiological study by Burch et al. [64] explored the link between brain tumor occurrence and accidents/injuries to the head or other parts of the body for 215 persons in Southern Ontario between 1979 and 1982. It was not specified if the head injuries were of the traumatic or non-traumatic variation. From their statistical analysis, no association was established between brain tumor formation and injuries. However, given the inclusion of other injury types besides those of the head, it is difficult to draw a direct comparison with our study. Colantonio et al. [5] showed that the predominantly urbanized region of Southern Ontario has a high rate of THI. Hence, the findings of our study can be useful for raising awareness and continual monitoring of THI as a potential causative factor in the development of brain cancer for the study region’s population.

Our analysis has also shown that being overweight or obese was associated with an increased risk of developing brain cancer. This finding was in agreement with previous epidemiological studies [15,16,65]. Benson et al. [65] explained that high EBF levels may cause cancers including malignant brain tumors through various biological mechanisms such as elevated inflammation and increased insulin resistance. In another observational study by Moore et al. [66], amongst 499,437 participants, the risk of glioma was nearly four times higher amongst obese adults than those with normal weight. Overall, obesity is considered to be an adverse prognostic factor for the increased risk of multiple forms of cancer including those of the brain [67]. However, a clear understanding of the obesity-related metabolic factors and mechanisms which link elevated BMI with cancer progression remains unclear [68]. To address this, future epidemiological studies investigating the risk of brain cancer due to obesity should also target exploration of inflammation levels, blood sugar levels, and other biological pathways which influence BMI. Our research adds further support to the notion that EBF is linked with heightened brain cancer risk. This knowledge is particularly important for health authorities in regions such as Southern Ontario where obesity is endemic [6].

We followed a probabilistic Bayesian regression approach for the space–time analysis of brain cancer risk. As opposed to a purely spatial analysis of the disease that is independent from one year to the next, the sequentially based spatio-temporal approach enabled us to study the geographical persistence and variation of the disease over multiple years across individual LHIN areal units. Furthermore, in comparison to frequentist-based approaches, the Bayesian approach enabled the modeling of unobserved factors through the use of spatial and temporal random effects, thus strengthening parameter estimation [69,70].

The accuracy of the data used in this study are also possibly influenced by various issues related to health care data collection such as under-reporting (i.e., cases of independent or dependent variables missed or not reported to LHIN health management systems) and lack of participation in health surveys. However, the Bayesian spatial smoothing used for our regression modeling handles such data sparsity-related problems, thus highlighting a benefit of the approach [71,72]. There are also possible practical applications of our work. Together with further research, the findings of this study can potentially be used for geographically targeted intervention of policies and decision-making related to management of the disease and its risk factors. For instance, the exceedance probability maps illustrated in Section 3 help to identify regions with elevated risk and this can be useful for disease surveillance and focused action by health authorities [21].

This study also had several limitations, including the need for longer-term data to enable further analysis of brain cancer evolution across the region. Such additional data can also be useful to aid in the confirmation of the association between brain cancer and the two evaluated risk factors. EBF information in this study was based on only one year of data and did not change over time. Given increased data availability for more years, future work should also analyze the effect of EBF on brain cancer when varying over both space and time. Further granular investigation is also needed to fully characterize the disease in the Southern Ontario region, including stratified analysis of different age, gender, and ethnicity groups.

Important potential unobserved/unmeasured covariates not included in the regression model can introduce ecological bias [21]. We consider this to be a general problem with observational studies. To cope with unobserved/unmeasured covariates in this study, we adopted the approach generally utilized in Bayesian disease mapping and analysis where, spatially structured and spatially unstructured random effects are included in the regression model [73]. For future work, we intend to explore other potential significant risk factor covariates such as age and gender which may be related to the outcome variable [2,62,74]. Other factors including socio-economic status have shown some association with brain cancer in previous work and may also be considered for future research [75].

The number of geographic units being used in this study has a direct impact on our results and analysis. This issue is related to the modifiable areal unit problem (MAUP) where the results (i.e., interpretation of statistical analysis and cartographic visualizations) obtained from analysing the aggregated data are dependent on the particular scale and boundaries used for the spatial analysis [76]. In addition to the LHIN regions, public health in the Southern Ontario region is also governed by Public Health Ontario who operates within 29 administrative boundaries (i.e., an increased number of geographic units compared to the LHINs) [77]. However, at the time of this study, the brain cancer count data used in our analysis were only available at the LHIN unit level [25]. Therefore, we note that: (i) it is important for future work to conduct analyses at finer spatial unit scales to confirm the risk factor associations highlighted by our study and (ii) there is every likelihood that different conclusions can be drawn/discovered at different geographic levels.

Another issue is the local nature of this study and the difficulty in generalizing the findings associated with brain cancer, THI, and EBF for other neighboring regions/provinces in Canada. As pointed out by Patel et al. [13], the majority of epidemiological studies are constrained to local geographic regions and specific time periods. To improve generalizability of results in a broader Canadian context, future work will require a combined multi-region analysis inclusive of other provinces in Canada. Comprehensive analysis across multiple time periods is also required to verify the impact of any potential significant risk factors which may be specific to the Canadian population at large.

## 5. Conclusions

This paper presented a study on the spatio-temporal analysis of brain cancer incidence in the region of Southern Ontario, Canada across a four-year period (2010–2013). To understand the geographical distribution and temporal variation of the disease in the 12 local health units of Southern Ontario, a Bayesian hierarchical regression modeling approach was utilized for brain cancer risk estimation. Specifically, a parametric space–time Poisson regression model was used to compute risk and its associated parameters, which included both fixed and random effects.

Our findings showed that the space–time variation of brain cancer risk ranged from 0.83 (95% credible interval (CI) 0.74–0.91) to 1.26 (95% CI 1.13–1.41) across all areal units from 2010–2013. Results also highlighted trends for disease risk progression during the four-year span. Overall, the mean trend for the study area smoothly decreased over time. However, 50% of the LHINs displayed area-specific trends which were higher than the region’s average, indicating a slower decrease in cancer rates for these spatial units in comparison to the mean trend. In addition, the findings of this research also suggest that severe head injuries and excess body fat levels are possibly associated with brain cancer risk in the Southern Ontario region. To gain further insights on brain cancer for the study region, future work will incorporate the use of additional data for an extended time frame along with the utilization of non-parametric spatio-temporal modeling approaches.

## Figures and Tables

**Table 1:** Summary statistics for study area data at LHIN level, Southern Ontario, Canada, 2010–2013.

	Minimum	Maximum	Mean	Standard. Deviation
LHIN Population	376,696	1,516,887	871,345	341,997
**Dependent Variable**				
Brain Cancer Count	30	140	80	30
**Independent Variables**				
Traumatic Head Injury (rate per 100,000 persons)	21.0	50.5	36.2	8.1
Excess Body Fat (%)	50.1	68.5	62.6	4.9

**Table 2:** Posterior distribution statistics for parameters of spatio-temporal regression model.

Parameters	Mean (95% Credible Interval)	Standard Deviation	Monte Carlo Error
α	0.066 (−0.014, 0.146)	0.041	0.00013
$\beta_{EBF}$	0.038 (−0.017, 0.094)	0.028	0.00008
$\beta_{THI}$	0.081 (0.027, 0.132)	0.027	0.00008
γ	−0.021 (−0.050, 0.007)	0.015	0.00005
$\sigma_{U}$	0.035 (0.013, 0.091)	0.021	0.00007
$\sigma_{V}$	0.030 (0.012, 0.070)	0.015	0.00005
$\sigma_{\delta }$	0.026 (0.012, 0.055)	0.011	0.00003

## Supplementary Materials

The following are available online at https://www.mdpi.com/2076-3271/7/12/110/s1, Figure S1: (a) LHIN-specific brain cancer counts (2010–2013). (b) LHIN-specific crude rates for traumatic head injuries (2010–2013). (c) LHIN-specific percentages for excess body fat levels (2013), Figure S2: Relative risk for brain cancer associated with spatially-based random effects ($U_{i}$ and $V_{i}$), Figure S3: Relative risk for brain cancer associated with temporally-based random effects (γ and $\delta_{i}$), Figure S4: Relative risk for brain cancer associated with both covariate fixed effects (THI and EBF), Figure S5: Relative risk of all LHINs with 95% credible interval (vertical lines) and posterior mean (midpoint) using Normal prior: β, γ~Norm(0, 0.1), Figure S6: Relative risk of all LHINs with 95% credible interval (vertical lines) and posterior mean (midpoint) using Gamma prior: $1/\sigma_{U}^{2},\;1/\sigma_{V}^{2},1/\sigma_{\delta }^{2}\sim Gamma\left(0.001,\;0.001\right)$, Figure S7: Relative risk of all LHINs with 95% credible interval (vertical lines) and posterior mean (midpoint) using Uniform prior: $\sigma_{U},\;\sigma_{V},\;\sigma_{\delta }\;\sim Unif\left(0,\;1000\right)$, Figure S8: Relative risk of all LHINs with 95% credible interval (vertical lines) and posterior mean (midpoint) using selected priors for final model: β, γ~Norm(0, 0.0001) and $1/\sigma_{U}^{2},\;1/\sigma_{V}^{2},1/\sigma_{\delta }^{2}\sim Gamma\left(0.5,\;0.0005\right)$.
