# Supplementary material for: Bayesian Space–Time Analysis of Brain Cancer Incidence in Southern Ontario, Canada: 2010–2013
Source: Med Sci (Basel). 2019 Dec 14;7(12):110. doi: 10.3390/medsci7120110 (PMC6950658; doi:10.3390/medsci7120110)
Supplement: Supplementary file 1 [file medsci-07-00110-s001.pdf]

medical sciences

MDPI

Article

Supplementary material:

Bayesian space-time analysis of brain cancer incidence in Southern Ontario, Canada: 2010-2013

Ravi Ancil Persad [1,*]

[1] Independent researcher, Toronto, Canada
* Correspondence: persad_ra@outlook.com

Received: date; Accepted: date; Published: date

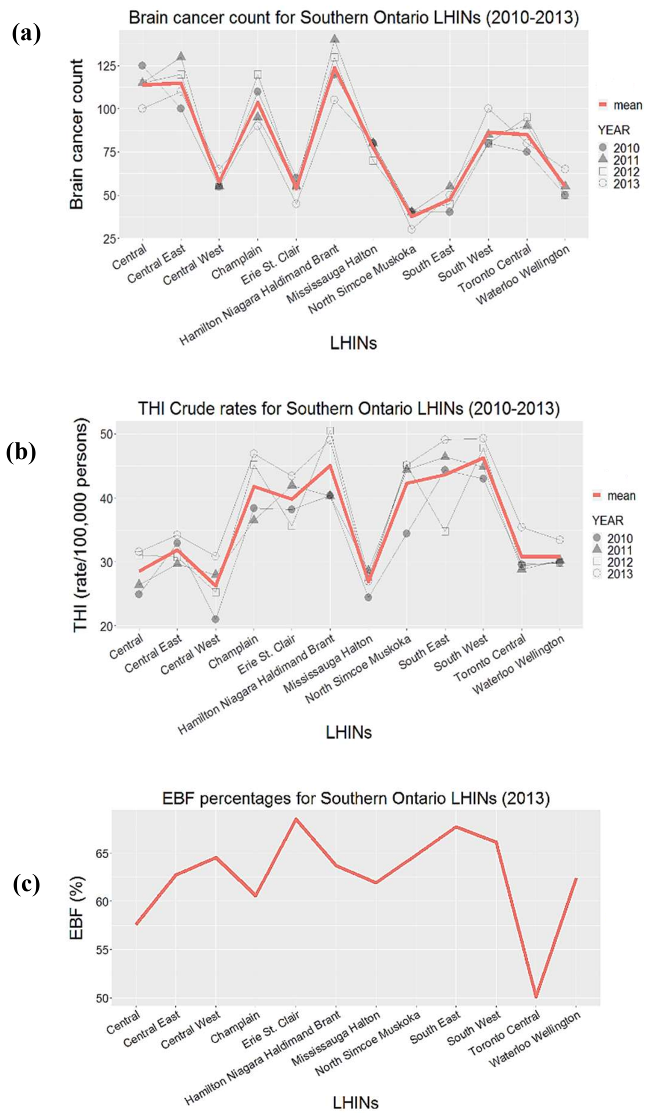

Supplementary Figure 1. (a) LHIN-specific brain cancer counts (2010-2013). (b) LHIN-specific crude rates for traumatic head injuries (2010-2013). (c) LHIN-specific percentages for excess body fat levels (2013).

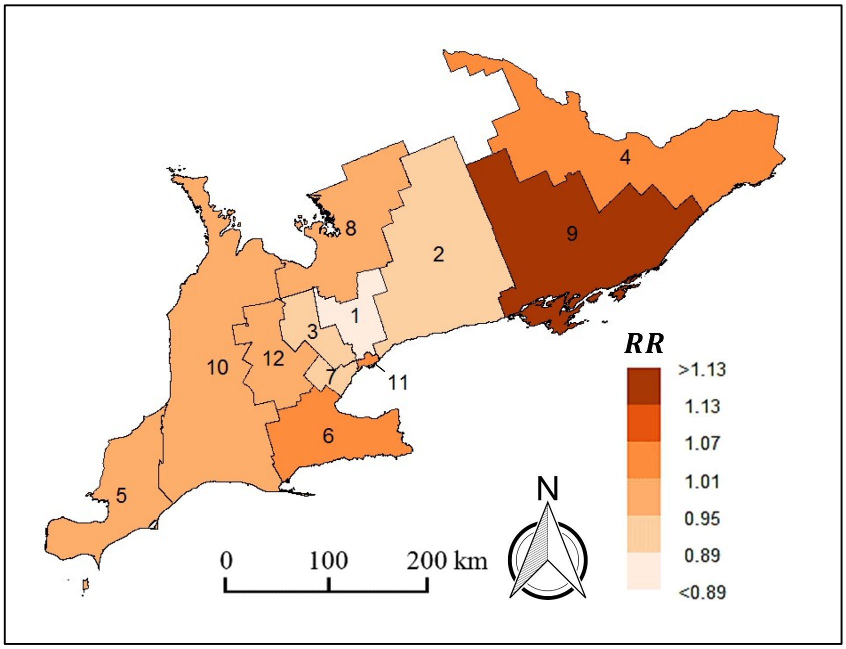

Supplementary Figure 2. Relative risk for brain cancer associated with spatially-based random effects (U_i and V_i).

NOTE: Explaining spatial relative risk patterns based on fixed and random effects

The relative risk for each LHIN is influenced by three main components: (i) the two covariate fixed effects (THI and EBF), (ii) the spatial structured and unstructured random effects, and (iii) the temporally-based random effects (i.e., the time trend and space-time interaction terms). We adapt the map decomposition approach of Law and Haining [35] to provide a visual interpretation on the contribution of these individual components toward the estimation of the overall relative risk over the assessed time frame. Upon visual inspection of the spatial patterns over each year, the overall relative risks shown in Figure 2 in the main manuscript were highly influenced by the covariate fixed effects (Supplementary Figure 4) in comparison to both the spatially and temporally-based random effects (Supplementary Figures 2 and 3). Further visual checks also show that the relative risk of the eastern LHINs (IDs. 4 and 9) are strongly driven by the spatially-based random effects (Supplementary Figure 2). The spatial distribution of the relative risk associated with the temporal random effects (Supplementary Figure 3) played a contributory role toward the overall determination of relative risk, however it did not dominate over the purely spatial random effects. Moreover, the contribution from temporal random effects were particularly weak in 2013.

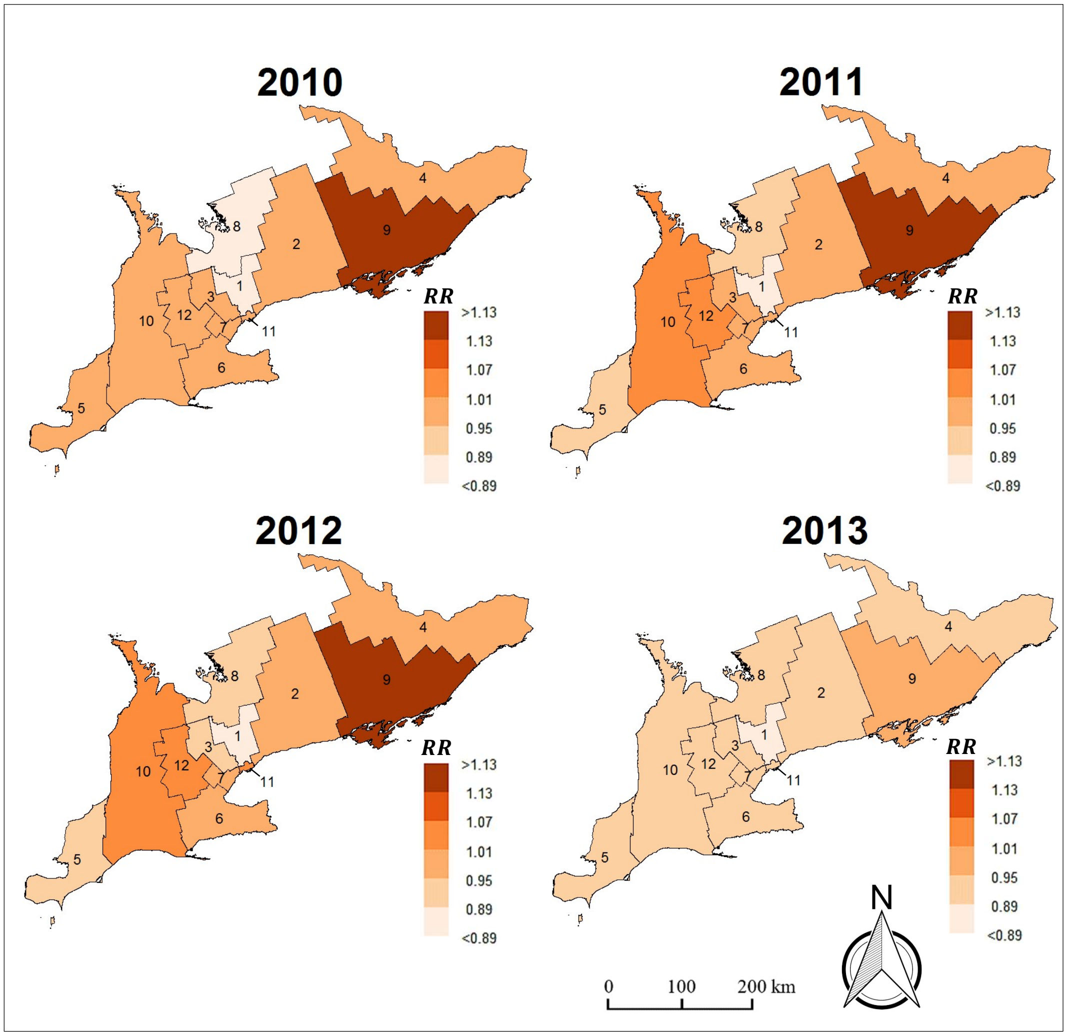

Supplementary Figure 3. Relative risk for brain cancer associated with temporally-based random effects (γ and δ_i).

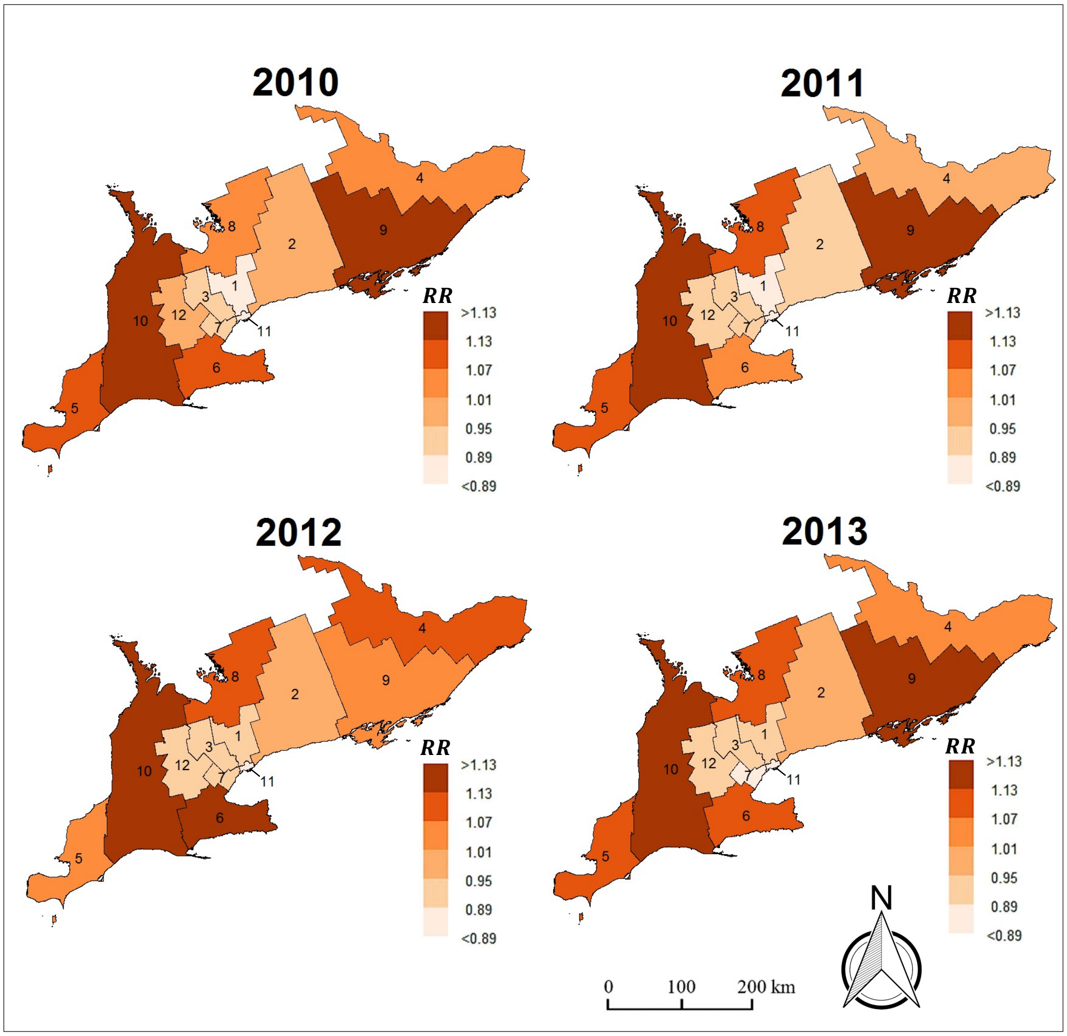

Supplementary Figure 4. Relative risk for brain cancer associated with both covariate fixed effects (THI and EBF).

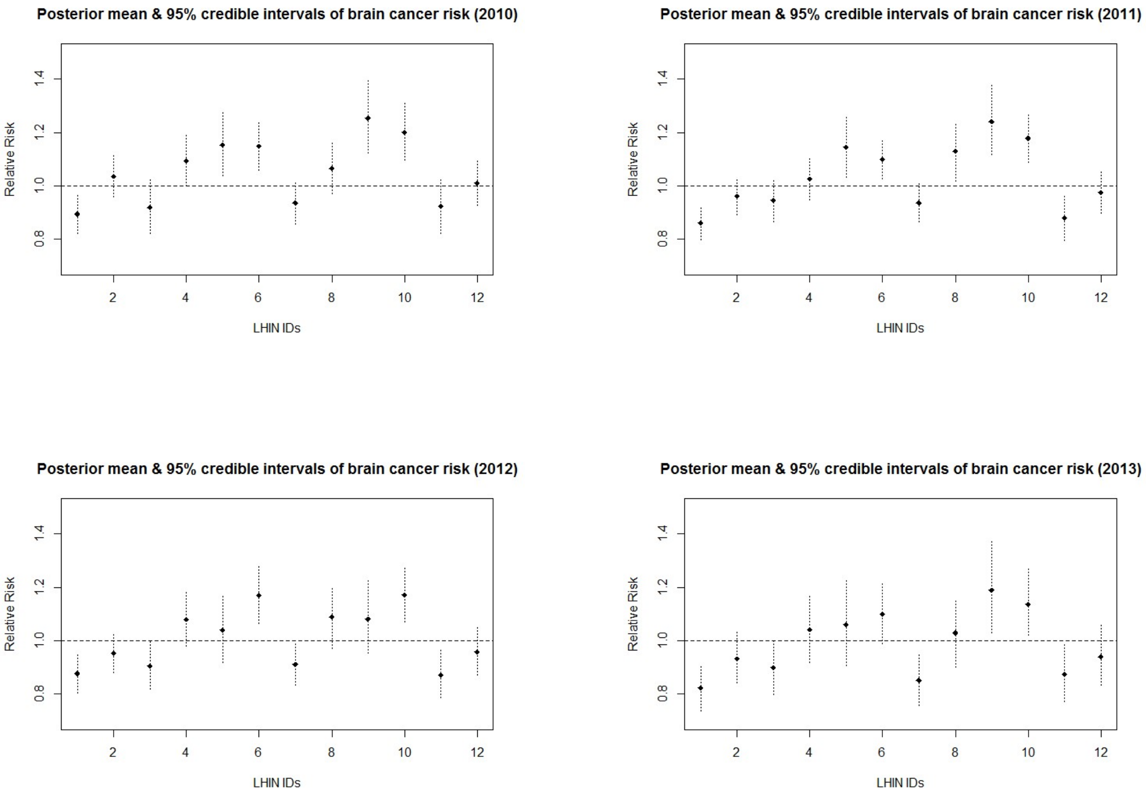

Supplementary Figure 5. Relative risk of all LHINs with 95% credible interval (vertical lines) and posterior mean (midpoint) using Normal prior: $\beta, \gamma \sim Norm(0, 0.1)$.

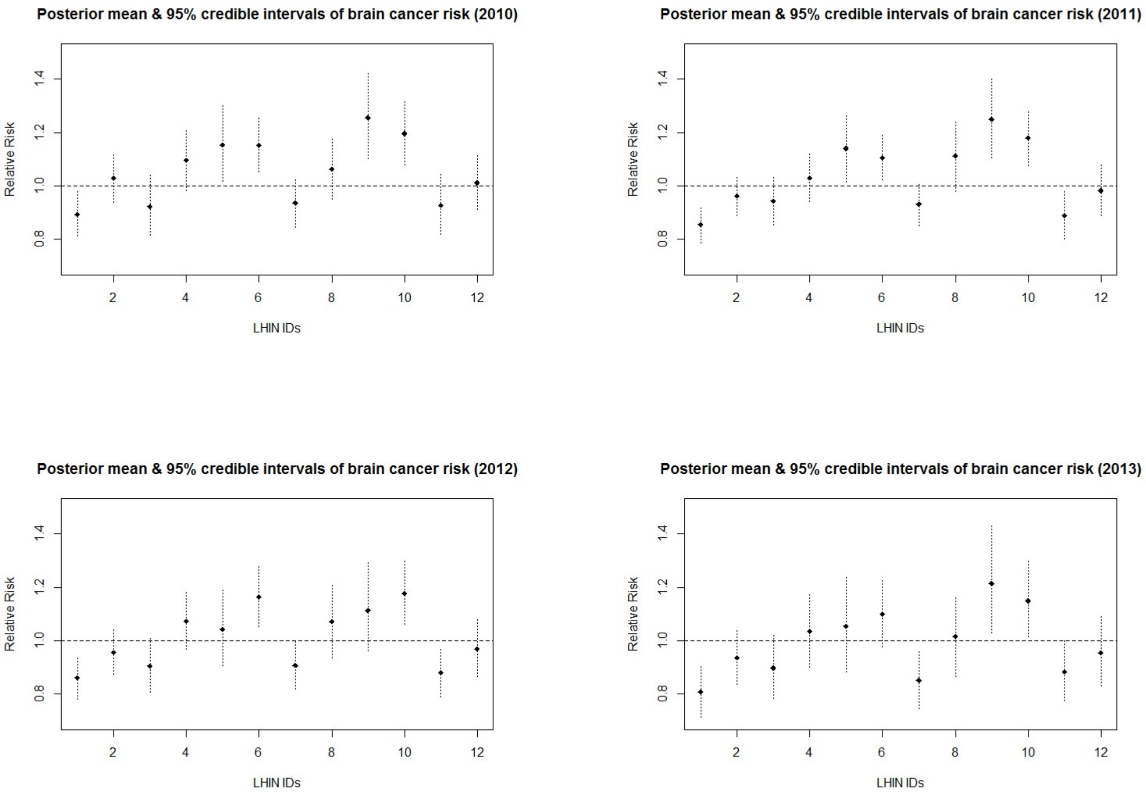

Supplementary Figure 6. Relative risk of all LHINs with 95% credible interval (vertical lines) and posterior mean (midpoint) using Gamma prior: $1/\sigma_U^2, 1/\sigma_V^2, 1/\sigma_\delta^2 \sim Gamma(0.001, 0.001)$.

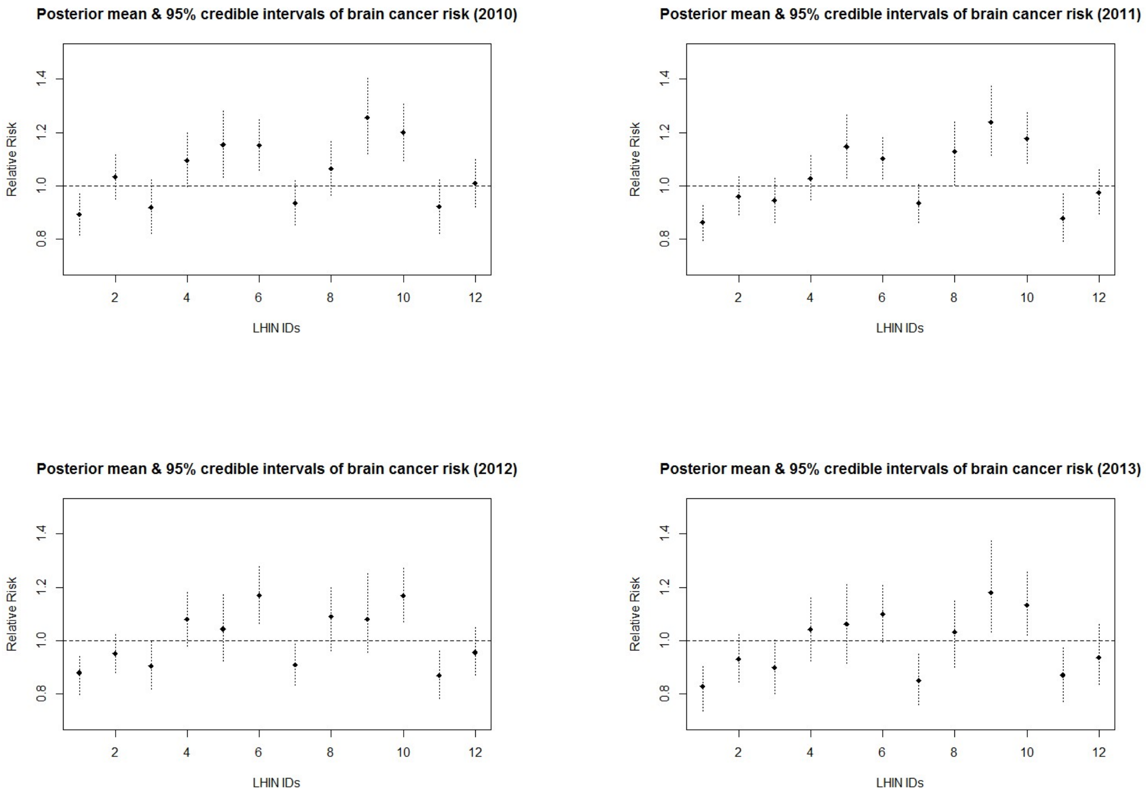

Supplementary Figure 7. Relative risk of all LHINs with 95% credible interval (vertical lines) and posterior mean (midpoint) using Uniform prior: $\sigma_U, \sigma_V, \sigma_\delta \sim Unif(0, 1000)$.

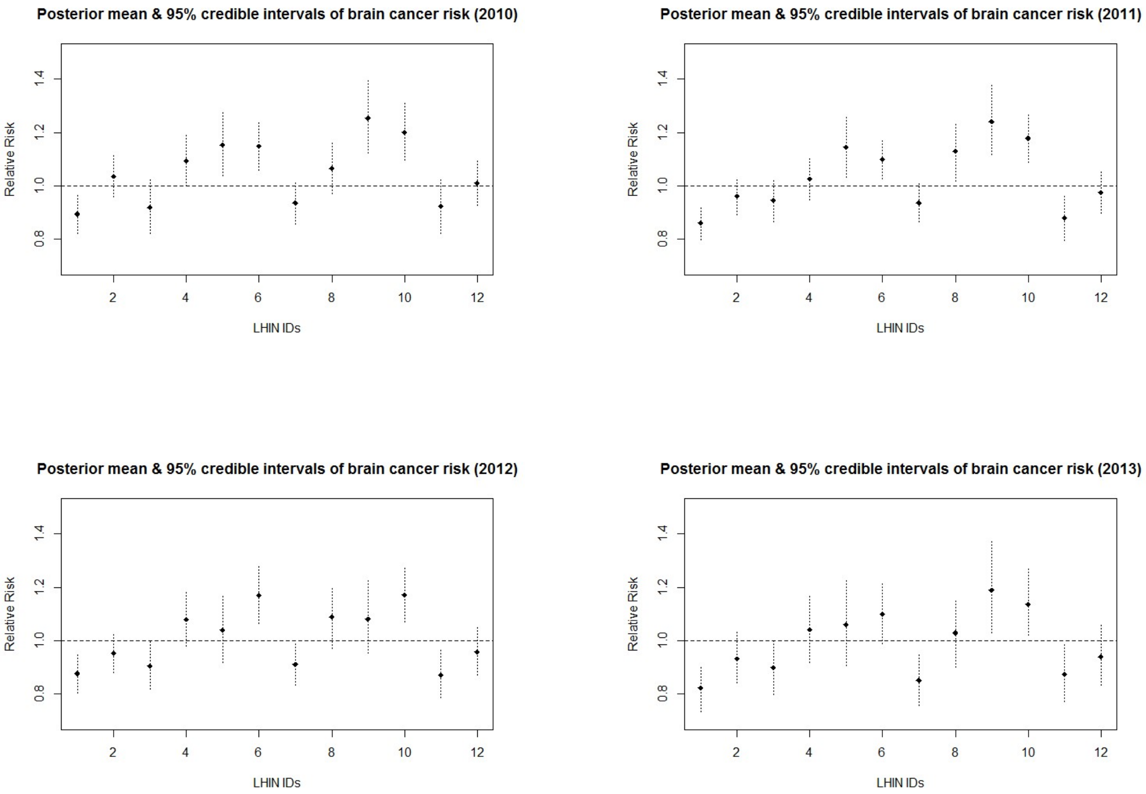

Supplementary Figure 8. Relative risk of all LHINs with 95% credible interval (vertical lines) and posterior mean (midpoint) using selected priors for final model: $\beta, \gamma \sim Norm(0, 0.0001)$ and $1/\sigma_U^2, 1/\sigma_V^2, 1/\sigma_\delta^2 \sim Gamma(0.5, 0.0005)$.
